# The effect of caffeic acid phenethyl ester on cell cycle control gene expressions in breast cancer cells

**DOI:** 10.22099/mbrc.2020.38811.1563

**Published:** 2021-03

**Authors:** Tuğçe Balc-Okcanoğlu, Sunde Yilma-Susluer, Cagla Kayabasi, Besra Ozme-Yelken, Cigir Biray-Avci, Cumhur Gunduz

**Affiliations:** 1 Vocational School of Health Sciences, Near East University, Nicosia, TRNC, Cyprus; 2 Faculty of Medicine, Department of Medical Biology, Ege University, Bornova, Izmir, Turkey

**Keywords:** CAPE, MCF-7 cell line, Cell cycle control genes

## Abstract

We aimed to find the effect of caffeic acid phenethyl ester (CAPE) on the expression profiles of cell cycle control genes in breast cancer cell line (MCF-7). The cytotoxic effect of CAPE on MCF-7 cell line was found with an XTT analysis. Total RNA was isolated from the cells exposed to IC_50_ dose and untreated control cells. Expressions of genes related to cell cycle control (*CCND2*, *RB1*, *ATM*, *CDC34*, *CDK5RAP1*) were evaluated by qRT-PCR by the LightCycler 480 System (Roche). *GAPDH* and *ACTB* housekeeping genes were used for the normalization of gene expressions. IC_50_ value of CAPE in MCF-7 cells was calculated as 75µM. It was shown that IC_50 _dose of CAPE induced significant upregulation in expressions of cell cycle control genes, compared to control cells. CAPE increases the expression of genes that are important in cell cycle control, suggesting that this component can be used as an effective chemopreventive agent in breast cancer cells.

## INTRODUCTION

Breast cancer is one of the most important causes of cancer-related deaths among women due to the high rate of metastasis [1]. Various compounds are used in conventional and alternative medicine. Caffeic acid phenethyl ester (CAPE) is one of the important components of propolis [2]. Its antioxidant activity or behavior as an inhibitor of enzymes and proteins indicate that CAPE has anti-apoptotic and anti-inflammatory effects. CAPE has been shown to be highly toxic in various studies. Its chemotherapeutic and chemopreventive effects have been studied in human cancer cell lines [3]. Several studies have found that CAPE has an effect on cell cycle progression, cell proliferation, cell cycle arrest and apoptosis [4]. CAPE can cause cell cycle arrest by inhibiting cyclin D and E proteins and c-Myc expression [5]. Cyclin D2 (CCND2) has significant functions in cell cycle regulation, differentiation and malignant transformation [6]. The retinoblastoma susceptibility gene (*RB1*) is known as a tumor suppressor. *RB1* mutations include all familial and sporadic forms of retinoblastoma. The RB gene is a common mutation in various human cancers [7]. ATM phosphorylates different proteins contained in cell cycle control points, apoptosis and DNA repair, including H2AX, p53, RPAp34, BRCA1, Chk2 [8]. In our study, the cytotoxic effect of CAPE on estrogen-dependent MCF-7 cells line and its effect on expression profiles of cell cycle control genes were investigated.

## MATERIALS AND METHODS

CAPE was obtained from Sigma-Aldrich (CAS Number: 104594-70-9). Stock solution was prepared with 0.1% dimethylsulphoxid (Biological Industries), and then diluted in cell culture medium. MCF-7 cell line was provided from ATCC (USA). MCF-7 cell line was cultured and maintained with RPMI 1640 Biological Industries (Kibbutz Beit-Haemek, Israel) medium containing 10% inactivated fetal bovine serum and 1% L-glutamine, 1% penicillin/ streptomycin Sigma–Aldrich (St Louis, MO, USA). Cell lines were maintained in a humidified incubator at 37°C with 5% CO_2_. Cell line was cultured in 37^0^C and 95% humidity with 5%CO_2_ incubator. The viability of the cells was founded by trypan blue dye exclusion test.

**Figure 1:**
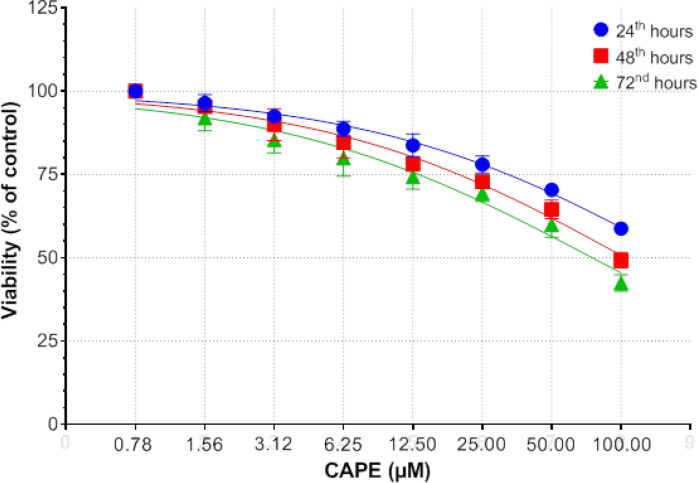
Cytotoxic effects after the implementation of CAPE

For determination of the IC_50 _dose, cells were seeded in 96-well plates and allowed to incubate for 24 hours. CAPE (dose dependent) inhibited cell growth on MCF-7 and human breast adenocarcinoma line cells MDA-MB-231 cell lines; but did not show any significant effect on normal cells [9]. Subsequently, CAPE (doses of 50 μM to 100 nM) was added. Cells were allowed to incubate at 24, 48 and 72 hours. The doses are shown on the Figure 1. XTT analysis was performed. Formazan formation was quantified spectrophotometrically at 450 nM-620 nM, with a microplate reader (Multiskan FC, Thermo Scientific, Vantaa, Finland). Viability was calculated by using the background-corrected absorbance.

Total RNA (Roche, Germany-High Pure RNA Isolation Kit) was isolated from cells exposed to IC₅₀ doses at 24, 48, 72 hours. The concentration and purity of RNA samples were determined by measuring absorbance at the wavelengths of 260/280 and 230/260 nm using a NanoDrop instrument (Thermo Scientific). 1‐5μg total RNA was transformed into cDNA with Transcriptor First Strand cDNA Synthesis Kit (Roche, Germany). For *CCND2*, *RB1*, *ATM*, *CDC34*, *CDK5RAP1* gene expression profiles, was extracted from the CAPE -treated and untreated MCF-7 cells (2×10^6^ cells/ml). 2 housekeeping (*GAPDH* and *ACTB*) were normalized according to gene expression. Primers were designed as span exon-exon junctions. SYBR Green PCR Master Mix and primers (Thermo Scientific, USA) were used for cell cycle expression profiles and reference gene analysis. 

Expression analyzes were performed by LightCycler® 480 real-time PCR. (Roche Life Science). Cytotoxicity analysis was performed with GraphPad Prism v5.0 Software. Data were analyzed by the “ΔΔCT method using“ Light Cycler® 480 Quantification Software. Statistical analysis was performed with Web-based RT2 ProfilerTM PCR Array Data Analysis.

## RESULTS AND DISCUSSION

CAPE inhibited cell proliferation occurred in a dose- and time-dependent manner. After 72 hours incubation with CAPE, the IC_50_ dose of CAPE in MCF-7 cells was 75μM (Fig. 1). It has been observed in the studies (reference 9) that it has no effect when looked at healthy cell lines The analysis of gene expression related with cell cycle revealed that IC_50_ concentrations of CAPE caused altered cell cycle gene expression levels compared to untreated controls in the MCF-7 cell line. Compared to the control cells, *CCND2*, *RB1*, *ATM*, *CDC34*, *CDK5RAP1* gene expressions increased 201.55, 62.03, 14.37, 62.03 and 8.66-fold, respectively (Table 1).

**Table 1:** Caffeic acid phenethyl ester (CAPE) Expression Analysis evaluation

Genes	Symbloes	Up-regulation	95% CI
Cyclin D2	*CCND2*	201.55	196.55-206.55
Retinoblastoma 1	*Rb1*	62.03	60.50-63.57
Cell Division Cycle 34	*CDC34*	14.37	14.01-14.73
ATM Serine/Threonine Kinase	*ATM*	62.03	10.92-11.47
CDK5 Regulatory Subunit Associated Protein 1	*CDK5RAP1*	8.66	8.45-8.88

Sanderson et al., looked at the activities of caffeic acid (CA) and CAPE on human androgen-dependent LNCaP prostate cancer cells. demonstrated that caffeic acid derivatives and CAPE have potent cytotoxic effects on LNCaP cell lines. Kabała-Dzik et al., administered doses of 10 to 100 µM at 24 and 48 hours to CA and CAPE activity on MDA-MB-231 cells. They found IC_50_ values for CAPE (depending on the dose) 27.84 µM (24 hours) and 15.83 µM (48 hours). They interpreted that it had low cytotoxic activity on MDA-MB-231 cells [10, 11]. 

CCND2 is a protein involved in cell cycle progression. It acts as the regulator of cyclin-dependent kinase (CDK) 4/6 in the G1-S transition. CDK4 (INK4) -retinoblastoma pathway inhibitor is CDK4 / 6 plays a critical role in cell cycle progression and contributes to endocrine therapy resistance in breast cancer [12]. *CCND2* is up-regulated in cell cycle arrest. Ectopic expression of CCND2 blocks the progression of the cell cycle. Lately, it has been determined that CCND2 regülation decreases in breast, lung and prostate cancers. CCND2 may act as an oncogene or tumor suppressor in various tumor types [6]. In the study directed by Ding et al., CCND2/3 was shown to important an oncogenic role. They showed high CCDN2 expression is associated with decreased overall survival in gastric cancer patients. On the contrary, high CCDN2 expression was found to be positive in non-small cell lung cancer patients. These findings suggest that CCND2/3 may be a therapeutic target and biomarker in various cancers [13]. CCND2 plays an integral role in cell cycle progression. Epithelial mesenchymal change (EMT) brings invasive, migration and stem cell properties to cancer cells and CCND2 down-regulates EMT [14].

The Rb1 pathway is known as the negative regulator of the cell cycle. RB1 losses have been observed in cancer cells, often associated with cell cycle control. Loss of Rb1 is important compared with many other cancer-related cellular processes, including differentiation. Down-regulation of miR-34c-3p results in high expression of *NCKAP1*, which inhibits cell proliferation in HCC cell lines and stops it in the G2/M phase by the RB1/P53 pathway [15].As a result of treatment with CAPE, *CCND2* and *Rb1* up‐regulation were evaluated to reduce cell proliferation and tumor growth in breast cancer. Following up-regulation of the ATM, cell cycle arrest and senescence occurred. Increased gene expression of ATM was considered to be positive. Increased CDC34 induced ubiquitin-mediated degradation and DNA replication of G1 regulators in the cell cycle [16].

Silencing of CDC34 could lead to the inhibition of cell proliferation and suppression of colony formation [17]. Increase of ches1 (Checkpoint suppressor 1) and *CDC34*, two genes related with the control point mechanism, was observed in a protected process in cell division to induce genomic stability and to induce cell cycle arrest in the G1 and G2 phase by DNA breakage [18]. In our study, the increase in the expression of *ATM*, *CDC34* was found to be significant. *ATM* and *CDC34* genes can contribute to cancer treatment as cell cycle regulators. A decrease in CDK5RAP1 was found to induce cell cycle arrest and apoptosis by ROS/JNK signaling pathways in MCF-7 cell lines. CDK5RAP1 is very important in the development of clinical treatments for cancer [19]. In addition, upregulated Cdk5rap1 is a specific CDK5 inhibitor involved in phosphorylating and activating the p53 transcription factor that promotes apoptosis [20]. The increase in expression of Cdk5rap1 is consistent with our study and may cause cell cycle arrest and apoptosis. CAPE increases the regülation of genes that are significant in cell cycle control, suggesting that this component can be used as an effective chemopreventive agent in breast cancer cells.
